# Supplementary material for: The Wheat Intrinsically Disordered Protein TdRL1 Negatively Regulates the Type One Protein Phosphatase TdPP1
Source: Biomolecules. 2025 Apr 28;15(5):631. doi: 10.3390/biom15050631 (PMC12109034; doi:10.3390/biom15050631)

## Slide 1
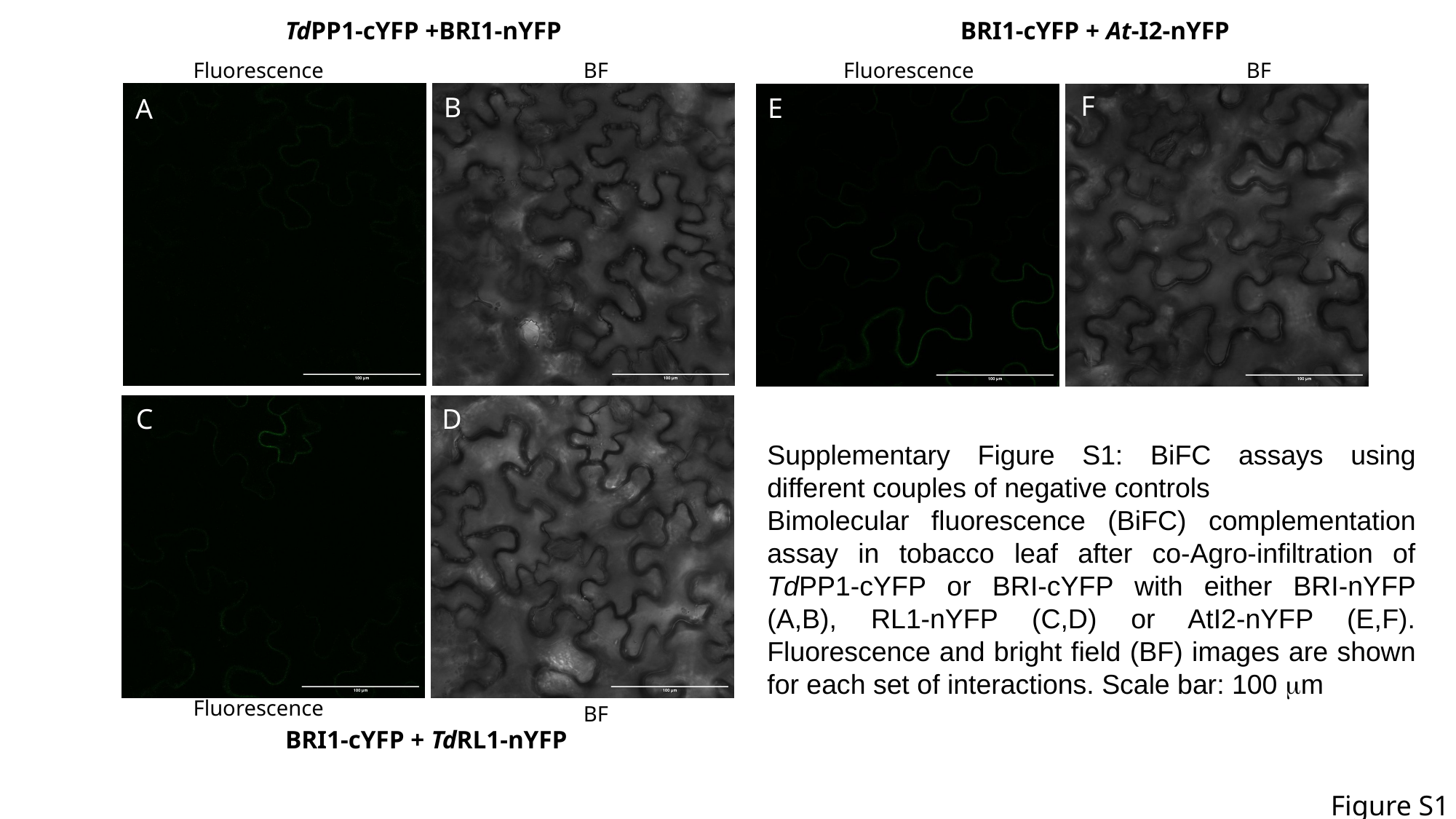

TdPP1-cYFP +BRI1-nYFP
BRI1-cYFP + At-I2-nYFP
Fluorescence
BF
Fluorescence
BF
F
B
E
A
C
D
Supplementary Figure S1: BiFC assays using different couples of negative controls
Bimolecular ﬂuorescence (BiFC) complementation assay in tobacco leaf after co-Agro-infiltration of TdPP1-cYFP or BRI-cYFP with either BRI-nYFP (A,B), RL1-nYFP (C,D) or AtI2-nYFP (E,F). Fluorescence and bright field (BF) images are shown for each set of interactions. Scale bar: 100 mm
Fluorescence
BF
BRI1-cYFP + TdRL1-nYFP
Figure S1

## Slide 2
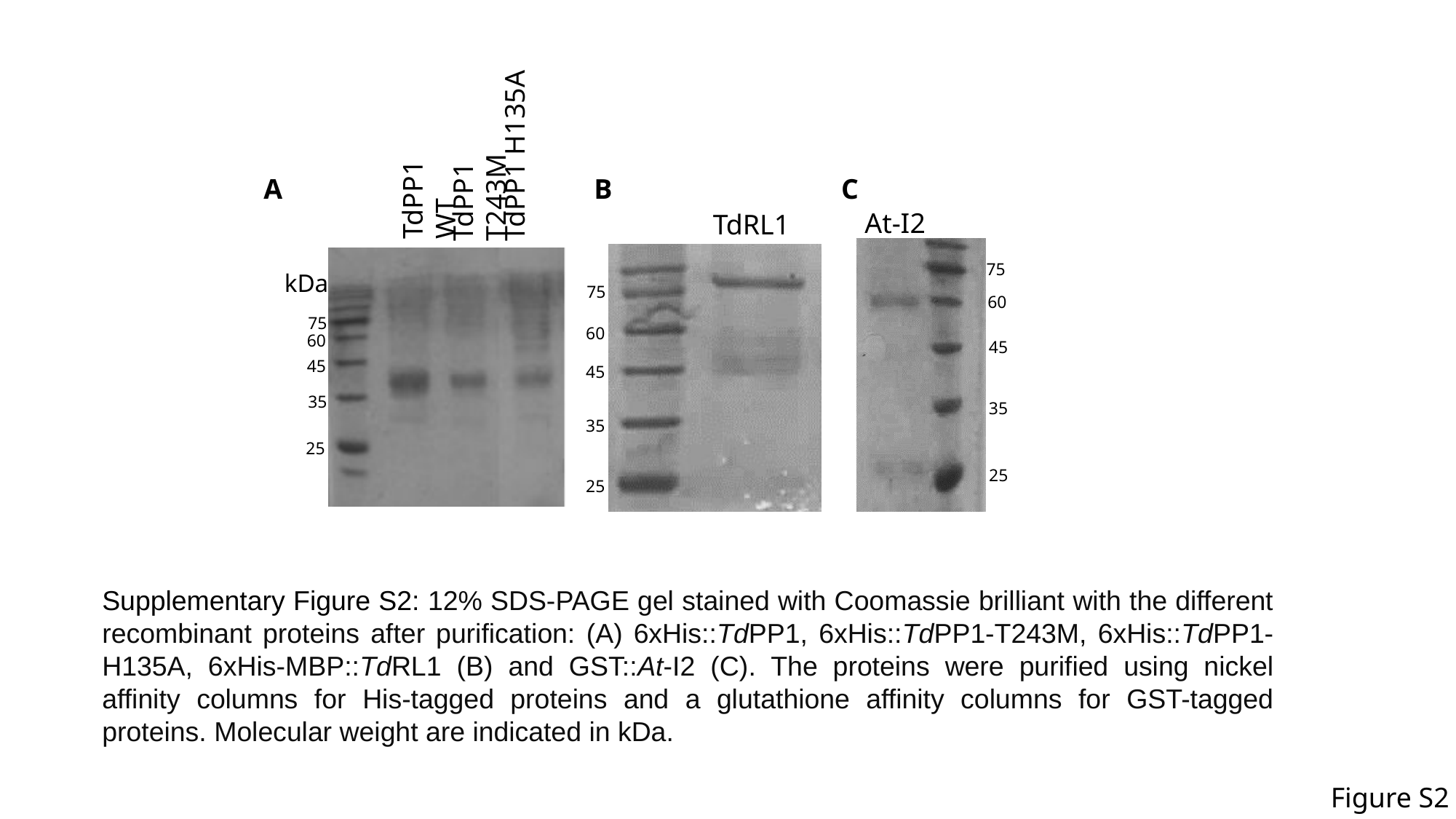

TdPP1 T243M
TdPP1 H135A
TdPP1 WT
A
B
C
At-I2
TdRL1
75
60
45
35
25
kDa
75
75
60
60
45
45
35
35
25
25
Supplementary Figure S2: 12% SDS-PAGE gel stained with Coomassie brilliant with the different recombinant proteins after purification: (A) 6xHis::TdPP1, 6xHis::TdPP1-T243M, 6xHis::TdPP1-H135A, 6xHis-MBP::TdRL1 (B) and GST::At-I2 (C). The proteins were purified using nickel affinity columns for His-tagged proteins and a glutathione affinity columns for GST-tagged proteins. Molecular weight are indicated in kDa.
Figure S2

## Slide 3
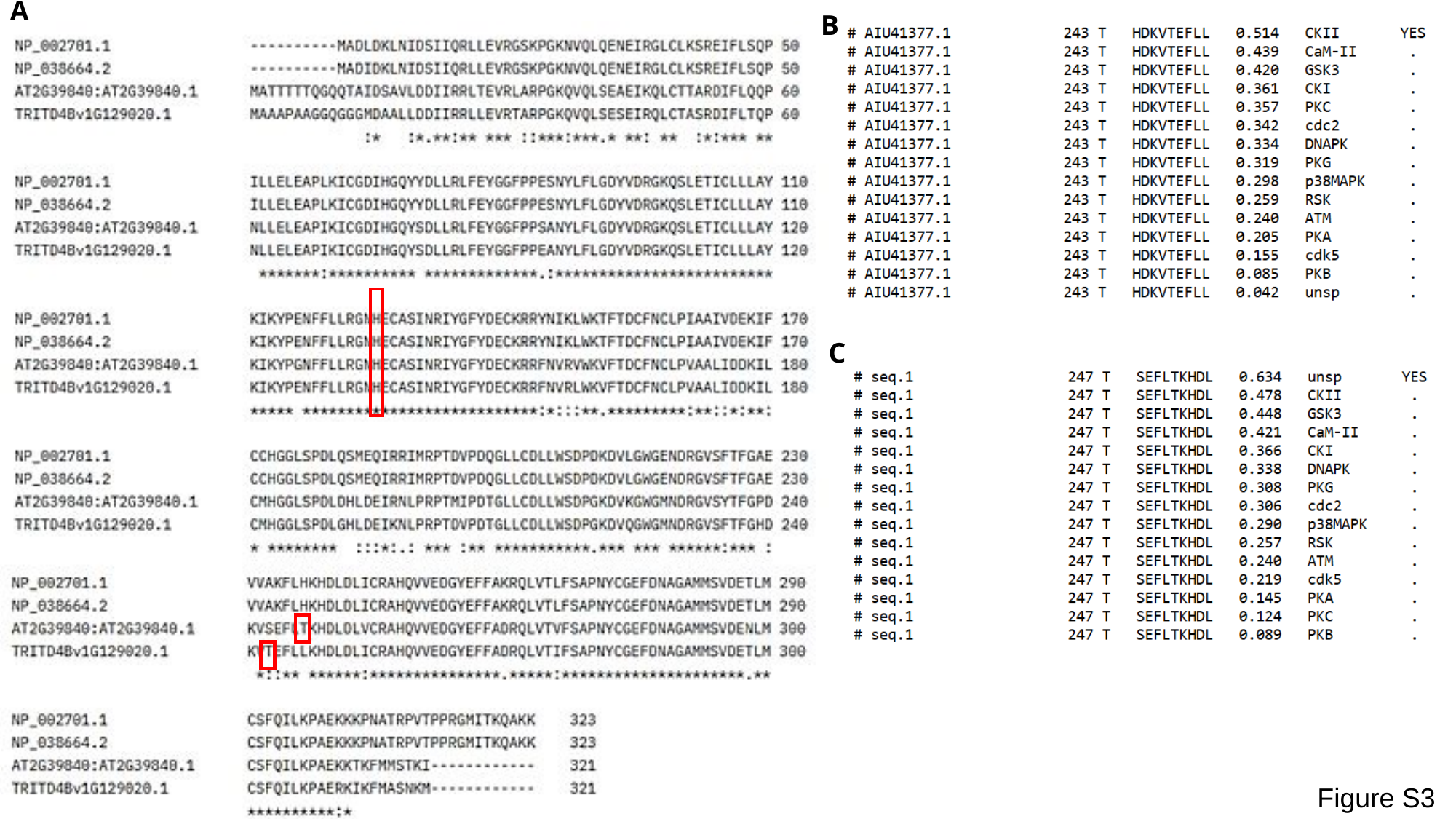

A
B
C
Figure S3

## Slide 4
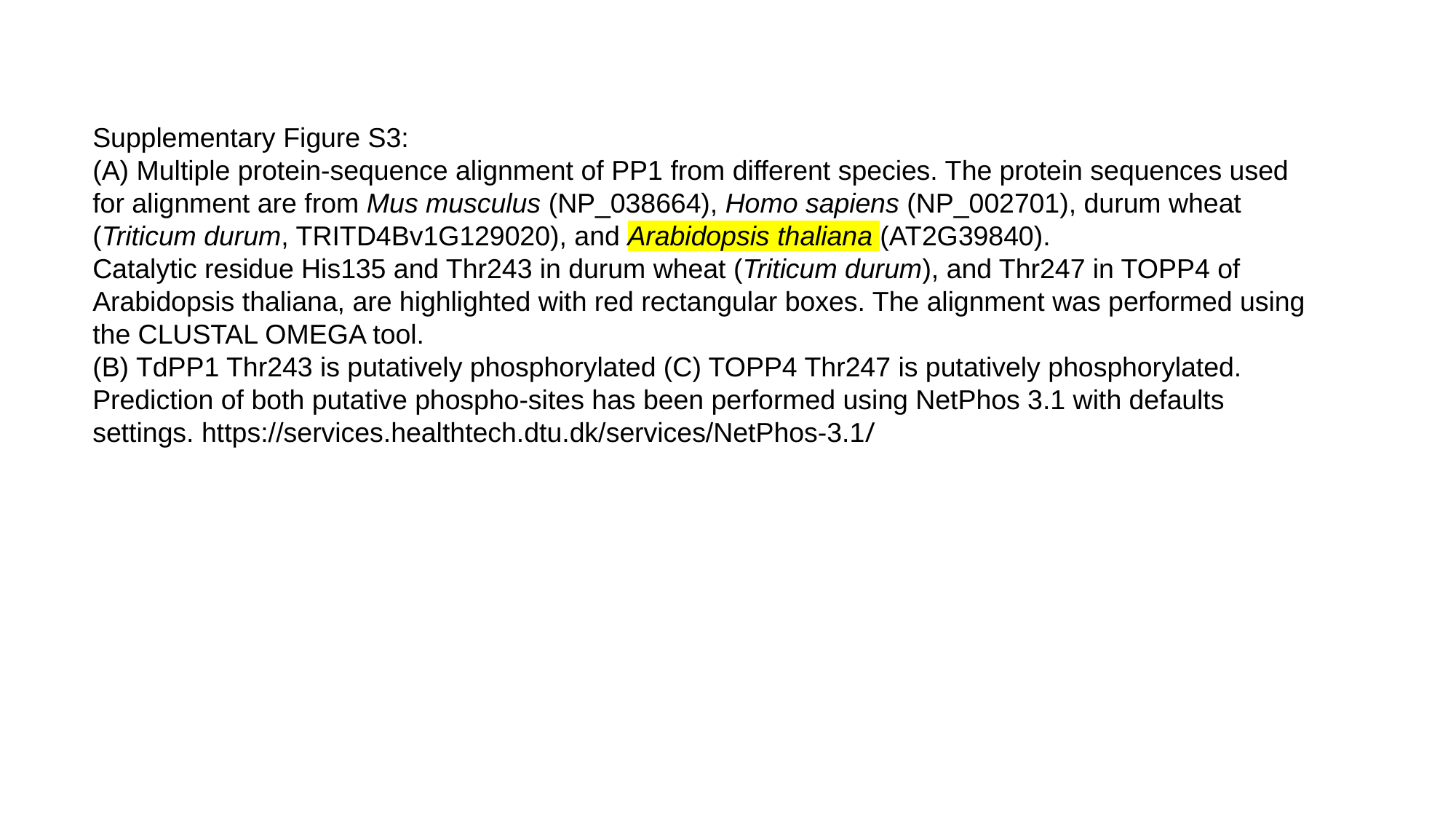

Supplementary Figure S3:
(A) Multiple protein-sequence alignment of PP1 from different species. The protein sequences used for alignment are from Mus musculus (NP_038664), Homo sapiens (NP_002701), durum wheat (Triticum durum, TRITD4Bv1G129020), and Arabidopsis thaliana (AT2G39840).
Catalytic residue His135 and Thr243 in durum wheat (Triticum durum), and Thr247 in TOPP4 of Arabidopsis thaliana, are highlighted with red rectangular boxes. The alignment was performed using the CLUSTAL OMEGA tool.
(B) TdPP1 Thr243 is putatively phosphorylated (C) TOPP4 Thr247 is putatively phosphorylated. Prediction of both putative phospho-sites has been performed using NetPhos 3.1 with defaults settings. https://services.healthtech.dtu.dk/services/NetPhos-3.1/

## Slide 5
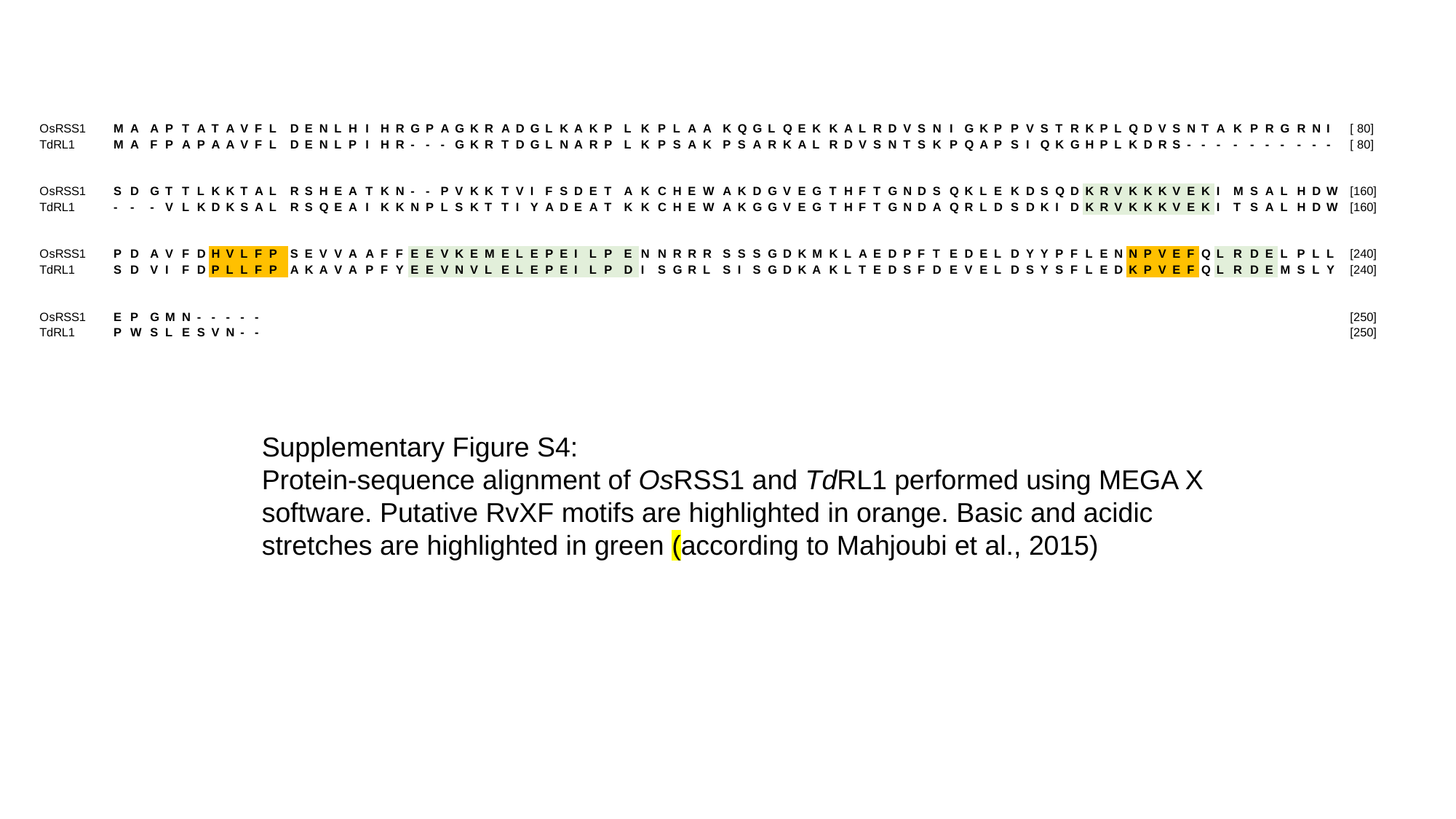

Supplementary Figure S4:
Protein-sequence alignment of OsRSS1 and TdRL1 performed using MEGA X software. Putative RvXF motifs are highlighted in orange. Basic and acidic stretches are highlighted in green (according to Mahjoubi et al., 2015)

## Slide 6
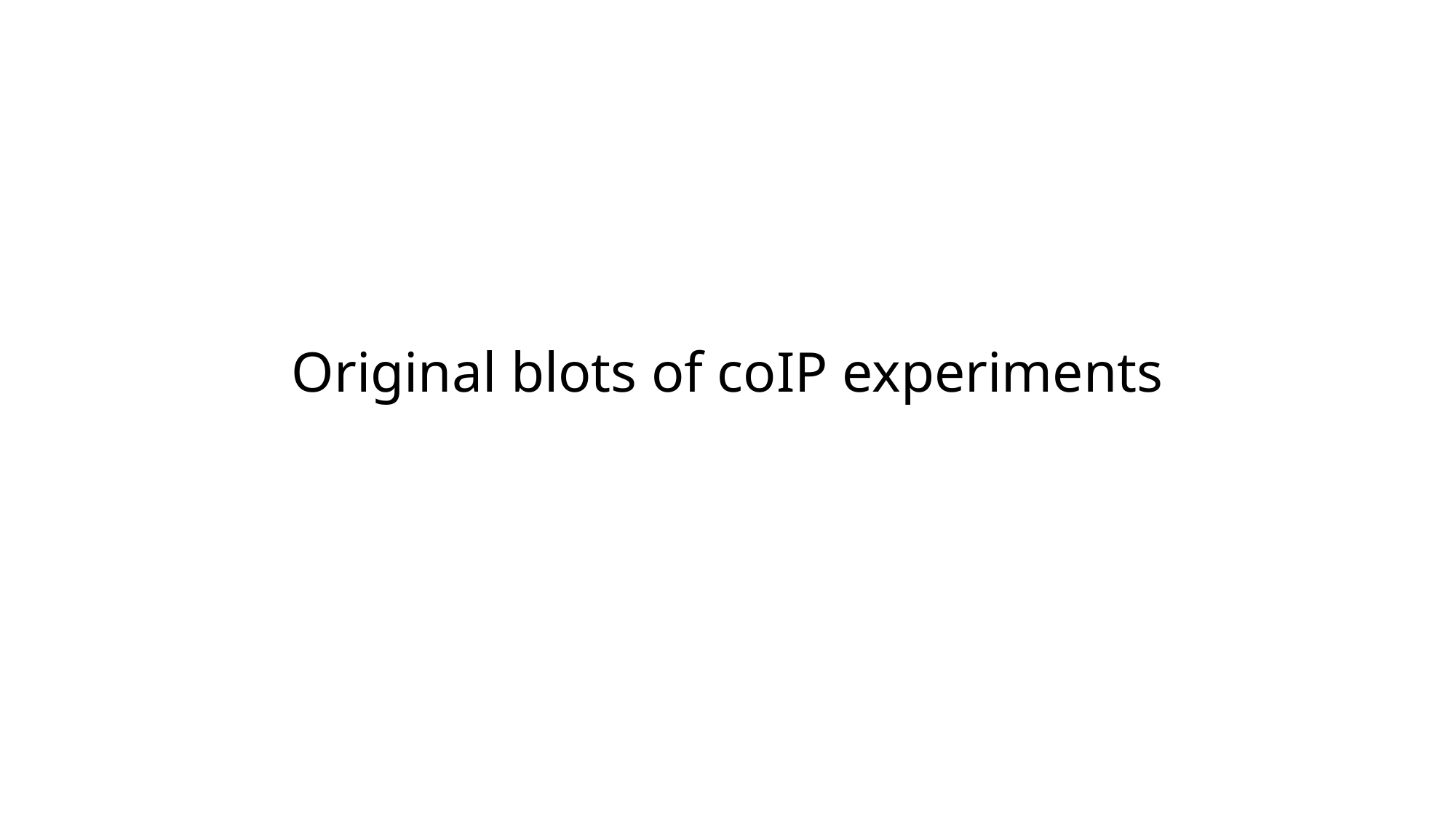

Original blots of coIP experiments

## Slide 7
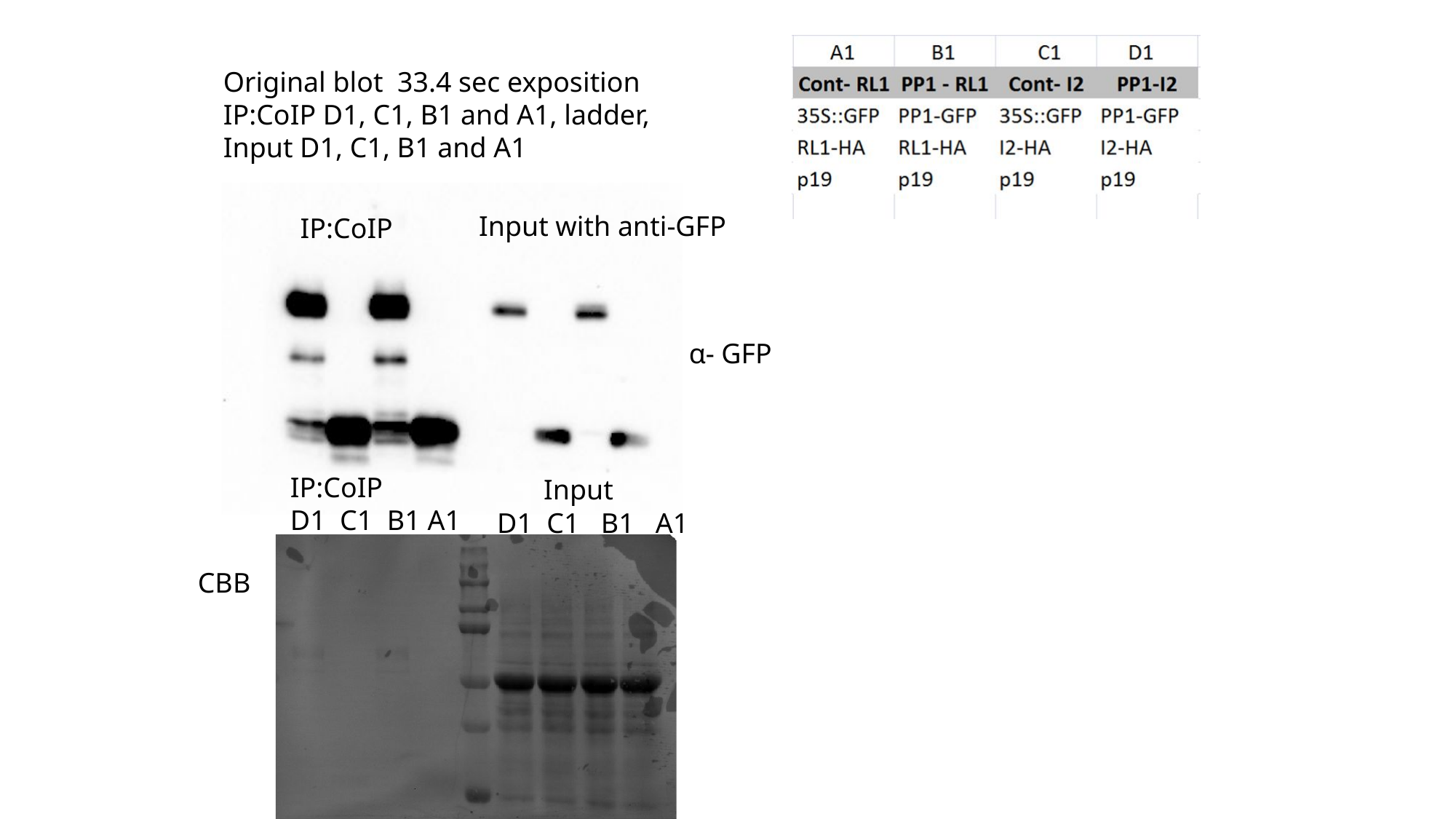

Original blot 33.4 sec exposition
IP:CoIP D1, C1, B1 and A1, ladder,
Input D1, C1, B1 and A1
Input with anti-GFP
IP:CoIP
α- GFP
IP:CoIP
D1 C1 B1 A1
Input
D1 C1 B1 A1
CBB

## Slide 8
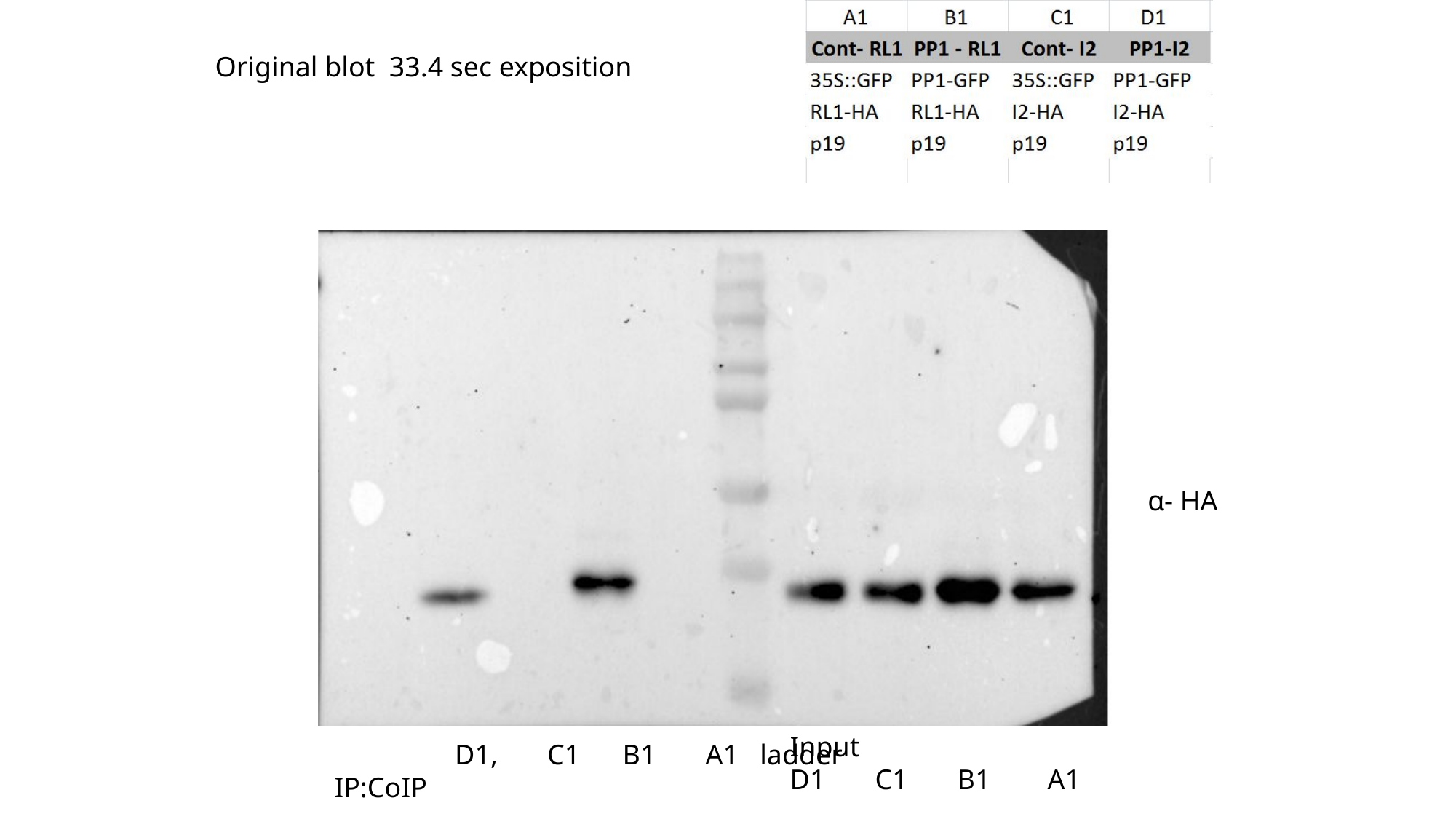

Original blot 33.4 sec exposition
α- HA
Input
D1 C1 B1 A1
 D1, C1 B1 A1 ladder
IP:CoIP

## Slide 9
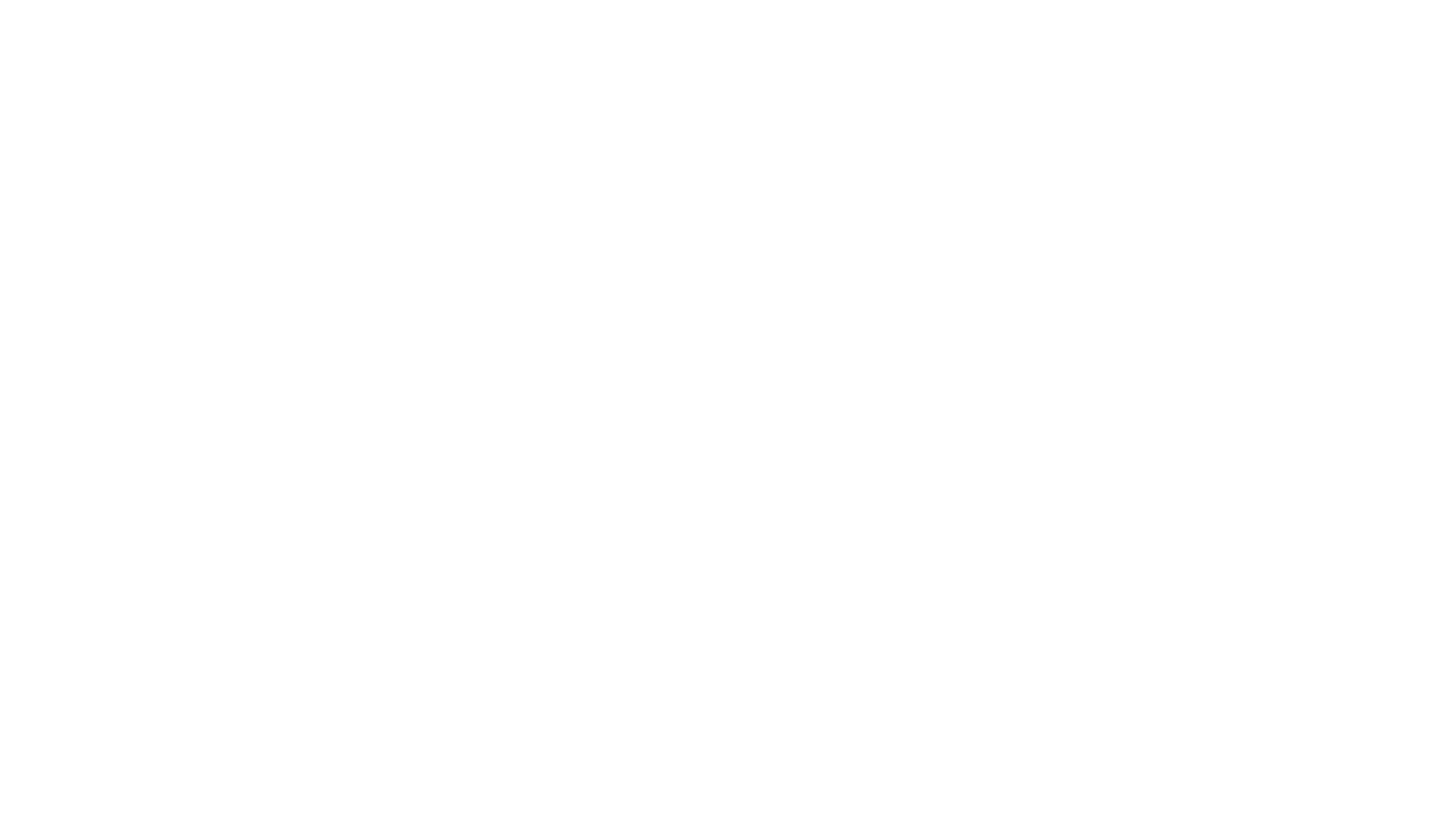

Supplement: Supplementary file 1 [file biomolecules-15-00631-s001.zip › biomolecules-3473878-supplementary.pptx]
